# Total-body [^18^F]FDG-PET/CT imaging of healthy volunteers with minimal effective dose

**DOI:** 10.1007/s00259-025-07644-x

**Published:** 2025-11-18

**Authors:** Daria Ferrara, Sebastian Gutschmayer, Zacharias Chalampalakis, Barbara Katharina Geist, Öykü Özer, Manuel Pires, Ivo Rausch, Werner Langsteger, Thomas Beyer

**Affiliations:** 1 QIMP Team, Medical University of Vienna, Vienna, Austria; 2 Division of Nuclear Medicine, Medical University of Vienna, Vienna, Austria

**Keywords:** [^18^F]FDG-PET/CT, Normal metabolism, Low-dose imaging, Effective dose

## Abstract

**Purpose:**

High-sensitivity, total-body (TB) positron emission tomography (PET) and computed tomography (CT) imaging systems enable substantial reduction of injected radioactivity without compromising image quality. Synthetic CT-like attenuation maps can be generated from PET data via deep learning (DL) to further minimise subject radiation exposure. We explored combining TB-PET with DL-derived attenuation maps to minimise effective dose in healthy subjects undergoing TB-PET/CT imaging with [^18^F]Fluorodeoxyglucose ([^18^F]FDG).

**Methods:**

47 healthy Caucasians (25 F/22 M, BMI: 24 ± 3 kg/m²) underwent TB-PET/CT imaging. After 6-hour fasting, subjects received low-dose CT (1 mSv) and (109 ± 7) MBq [^18^F]FDG, followed by a 62-minute dynamic PET acquisition (supine, arms down). PET data from 57 to 62 min were down-sampled to simulate reduced activities (50%, 25%, 10%, 5%). Effective doses (ED) were estimated for each activity level. Synthetic CTs (ED = 0 mSv) were generated from PET raw data (at all activity levels) and used to reconstruct attenuation-corrected PETs, which were compared to the original images. Organ-level segmentation enabled quantification of Standardized Uptake Values normalised to body weight (SUVbw) and coefficients of variation (CV).

**Results:**

Across the cohort, organ-based SUVbw differences remained < 10% versus reference PET for simulated activities down to 10%. At 25% activity (~ 25 MBq, ED~ 0.45 mSv), PET quantification remained robust, though CV increased in skeletal muscle and fat. At 5% activity, SUVbw deviations exceeded 10% in several organs.

**Conclusion:**

Total-body [^18^F]FDG-PET/CT enables reliable organ-level quantification (%-differences < 10%) at injected activities as low as ~ 25 MBq. Such low-dose protocols may support the creation of reference datasets of healthy controls while minimising radiation exposure to subjects and staff.

**Graphical Abstract:**

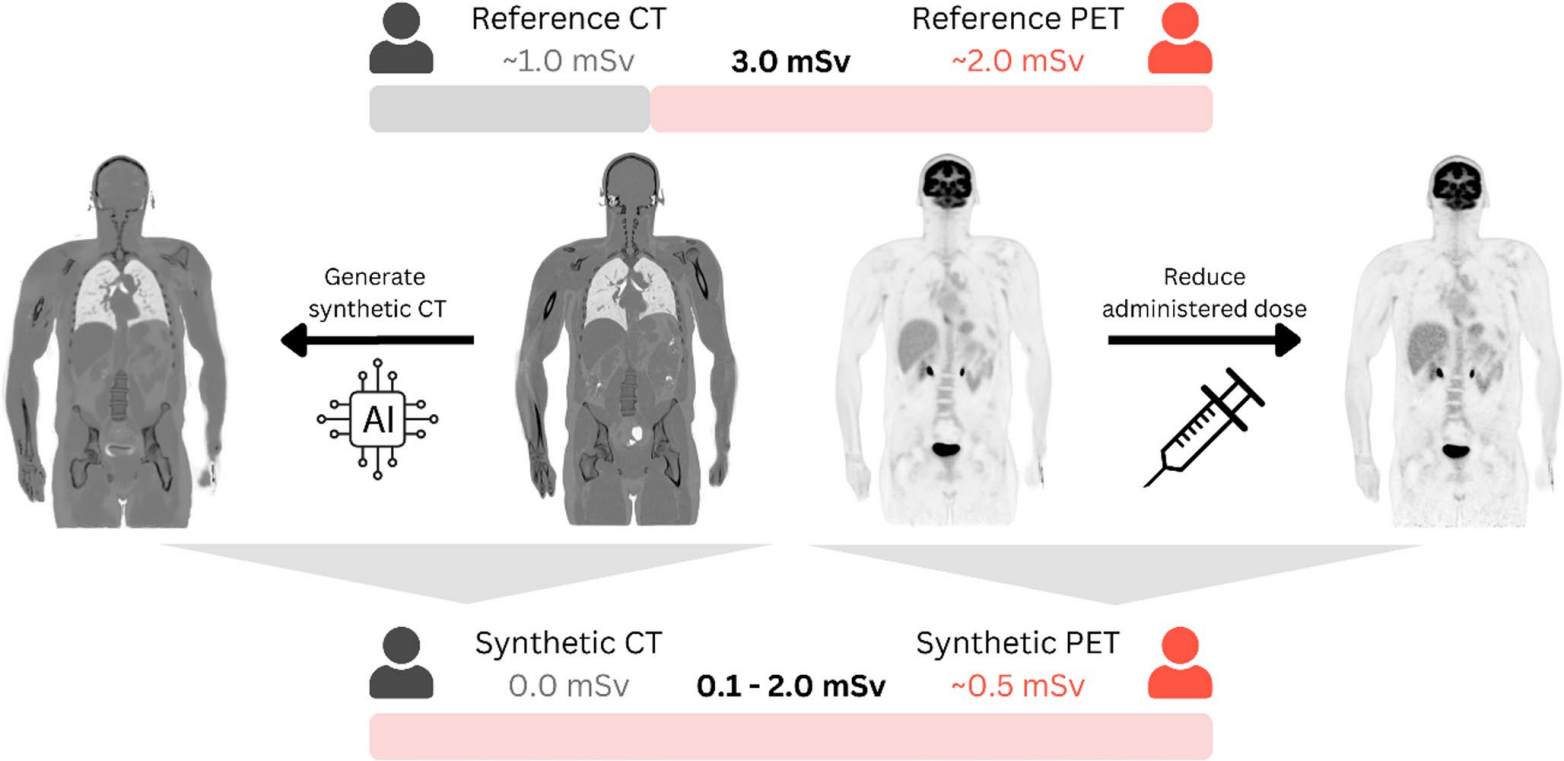

**Supplementary Information:**

The online version contains supplementary material available at 10.1007/s00259-025-07644-x.

## Introduction

Positron emission tomography combined with computed tomography (PET/CT) is a powerful dual-modality imaging method that provides both anatomical and physiological tracer-specific information. Since its inception in 2000 [1], growing evidence attests a key role of PET/CT imaging in combination with [^18^F]-labelled glucose analogue (Fluorodeoxyglucose, [^18^F]FDG) for evaluating physiological and pathological processes across various clinical fields. While primarily used in oncology for tumour detection, staging, and treatment monitoring [2–4], [^18^F]FDG-PET/CT has recently gained attention for its potential to characterise systemic metabolic patterns in cancer patients. This capability may pave the way for whole-body disease phenotyping [5], but it requires a reference metabolic standard - a “normative database” - against which disease-related deviations can be meaningfully interpreted.

Some studies have attempted to define such a baseline using unaffected organs from cancer patients [6, 7]; however, an ideal reference should be based on truly healthy subjects. Alternatively, normative datasets could be derived from PET/CT screening programmes of asymptomatic and presumably healthy subjects, as has been done in Japan [8] and China [9]. However, the value of PET-based screening, or surveillance imaging, in these populations remains controversial [10–12], and exposing asymptomatic subjects to ionizing radiation without a clinical indication is generally discouraged in most Western countries. In Europe, Diagnostic Reference Levels (DRLs) provide benchmarks for clinical [^18^F]FDG-PET/CT. For adults, these correspond to effective doses of about 5–7 mSv for the PET component and an additional 5–8 mSv for the CT, which corresponds to ~ 15 mSv in total when a fully-diagnostic CT is performed [13, 14]. Thus, conventional PET/CT protocols can result in 10–20 mSv of effective dose. Against this, exploring further dose reductions in healthy volunteers, even below already low-dose reference protocols, is consistent with the ALARA (As Low As Reasonably Achievable) principle and of particular relevance to studies aiming at normative datasets.

Recent technological advances in PET/CT imaging systems, namely the commercial introduction of total-body, or large axial field-of-view systems, such as the Siemens Biograph Vision Quadra [15, 16] and United Imaging uEXPLORER [17], provide significantly increased volume sensitivities [18] compared to previous-generation PET systems. This allows high-quality imaging with radiotracer doses potentially 10-fold lower [19] than used with conventional whole-body PET/CT systems [20, 21]. In parallel, AI-driven methods enable further dose reduction through noise-reduction techniques [22, 23]. Efforts have also been made to reduce the CT-related dose exposure, which often contributes the largest fraction of total exposure (3–15 mSv) [21]. To address this, emerging AI-based solutions like “CTlessPET” [24] can generate synthetic CT images directly from non-attenuation-corrected [^18^F]FDG PET images, potentially eliminating CT-related exposure. If the CTlessPET approach were to be combined with the very low-dose PET imaging protocols that become tenable with TB-PET, then radiation concerns over the use of TB-PET/CT for imaging healthy subjects could be addressed, and valuable information on normative tracer distributions could be obtained [25].

In this study, we aim to determine the minimum effective dose to healthy subjects undergoing total-body [^18^F]FDG-PET/CT imaging with synthetic CT-based attenuation correction while preserving the quantitative accuracy of the PET(/CT) readouts. If proven accurate, such imaging protocols could facilitate the creation of a normative metabolic [^18^F]FDG atlas to support future applications in disease characterisation and preventive imaging [26–28].

## Materials and methods

### Participants and Image Protocol

**Fig. 1:**
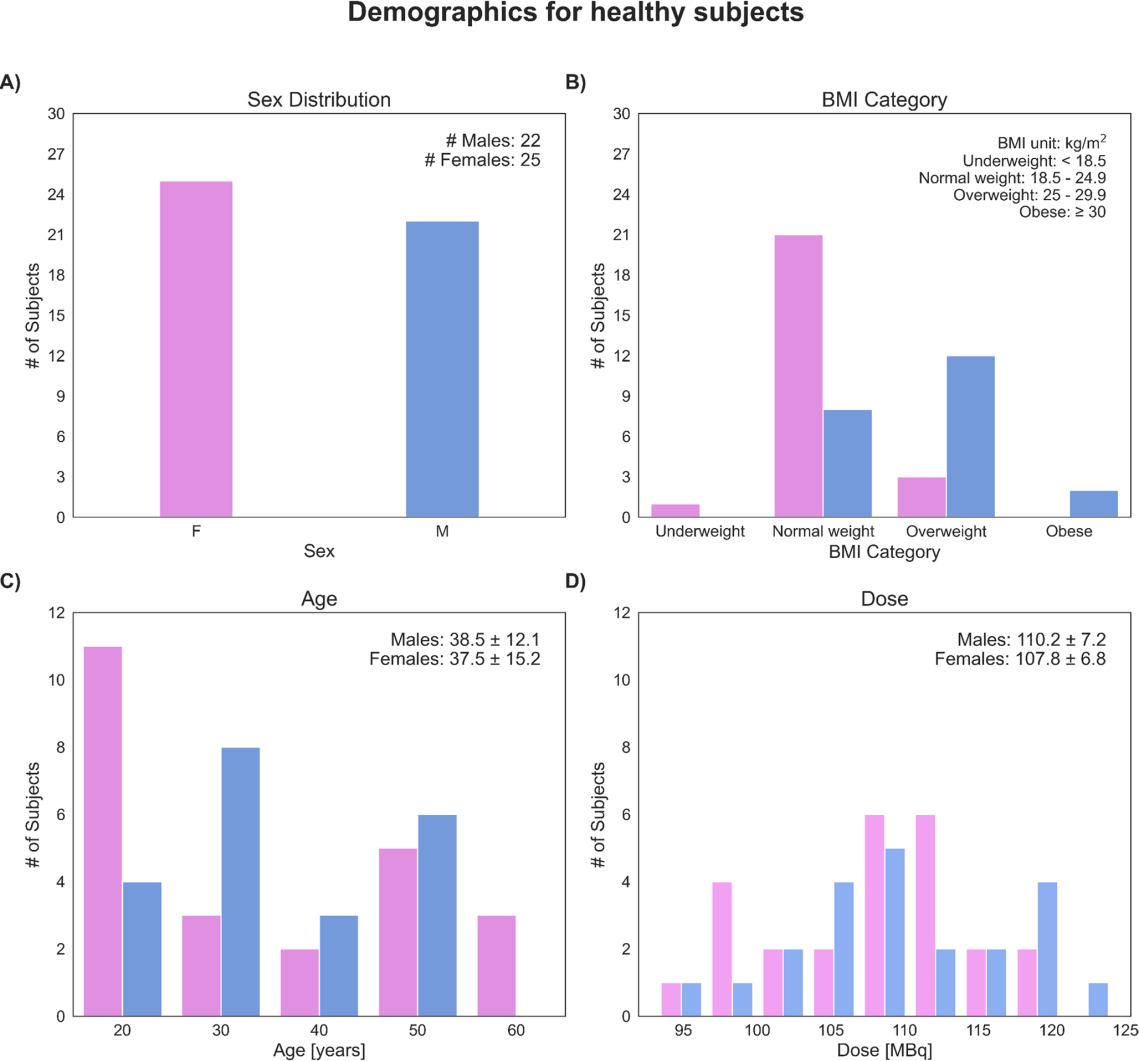
Demographics of the study participants. All subjects were healthy and of Caucasian ethnicity. (**A**) Sex distribution was balanced, with 47% male and 53% female participants. (**B**) Most individuals fell within the normal weight range (61.7%), based on BMI categorisation, with a notable prevalence of overweight males (26%). (**C**) Age distribution was imbalanced, with a majority of participants aged between 20 and 40 years. (**D**) The injected activity (labelled as "Dose") followed a Gaussian distribution, with a mean of 109 ± 7 MBq

This analysis includes [^18^F]FDG-PET/CT data of 47 healthy Caucasian controls: 25 (53%) females and 22 (47%) males. Mean age was 38 ± 14 years (range: 19–65 years). Demographic details are summarised in Fig. 1. Imaging data were acquired at the Medical University of Vienna, Austria, between September 2023 and August 2024 in accordance with the Declaration of Helsinki and approved by the local Ethics committee (IRB: EK 1707/2022) [25].

Imaging was performed on a Siemens Biograph Vision Quadra system (software VR20B), with subjects fasting for 6 h and positioned head-first-supine (arms down) during the 62-minute examination. The PET system features a 106 cm axial FOV, a spatial resolution of 3.3 mm (transverse) and 3.8 mm (axial) at a 1-cm offset, a sensitivity of 180 kcps/MBq, and a time-of-flight (TOF) resolution of 215 ps [15, 16].

### Reference PET/CT Acquisition

A low-dose spiral CT scan was acquired using automatic tube current modulation (25 mAs reference at 140 kVp), with tin filtration and a Br32f convolutional kernel, providing 1.523 mm × 1.523 mm in-plane resolution, and 2 mm slice thickness (512 × 512 × 531 voxel matrix). Images were reconstructed using the iterative ADMIRE algorithm (780 mm reconstruction diameter) and served as CT reference (refCT) for subsequent analysis and attenuation correction of the reference PET (refPET) images.

For the PET acquisition, subjects received an intravenous injection of 109 ± 7 MBq [^18^F]FDG. 62 min of list-mode emission data were collected as part of a dynamic study assessing test–retest variability and potential correlations between wearable readouts and FDG uptake in healthy controls [25]. Although current EANM guidelines [20, 29] recommend higher injected activities for [^18^F]FDG PET/CT, the high sensitivity of the Quadra system operated in Ultra-High Sensitivity (UHS) mode allowed a reduction to ~ 100 MBq without loss of diagnostic image quality. This activity level corresponds to a standard clinical examination of 2 min/250 MBq [^18^F]FDG on the same PET/CT system [30], or an 8 min/250 MBq [^18^F]FDG scan on a Biograph Vision 600 [16, 31]. PET images were reconstructed at 57–62 min post-injection (p.i.) with OP-OSEM with TOF/PSF corrections (4 iterations, 5 subsets; 440 × 440 × 531 matrix, 1.65 mm × 1.65 mm × 2 mm voxel spacing) in UHS mode (maximum ring difference = 322). A 2 mm FWHM Gaussian post-reconstruction filter was applied with standard corrections (attenuation, scatter, randoms, decay, and dead time).

The effective dose (ED) was calculated individually for each subject as the sum of the CT and PET contributions. For the CT component, the dose-length product (DLP), which is subject to the individual tube current modulation, was extracted from the scanner-generated report and multiplied by the conversion factor $\:\:k=0.015\:\frac{mSv}{mGy}\times\:cm$, as recommended for whole-body low-dose CT scans [32], resulting in approximately 1 mSv per subject:

1 $$\:E{D}_{CT}=DLP\:\left[mGy\times\:cm\right]\times\:k\:\left[\frac{mSv}{mGy}\times\:cm\right]\:$$

For the PET contribution, the injected [^18^F]FDG activity was multiplied by the dose coefficient $\:\varGamma\:=0.017\:mSv/MBq$ [33], yielding approximately 2 mSv:

2 $$\:E{D}_{PET}=Activity\:\left[MBq\right]\times\:\varGamma\:\:\left[\frac{mSv}{MBq}\right]$$

These values were subsequently adjusted for each individual’s body mass index (BMI) to determine the weight-scaled PET effective dose ($\:{ED}_{PET-BMI}$), as proposed by Willowson et al. [34]:

3 $$\:E{D}_{PET-BMI}=\frac{E{D}_{PET}\:\left[mSv\right]}{\sqrt{\frac{BMI\:[kg/{m}^{2}]}{22\:[kg/{m}^{2}]}}}$$

The total effective dose was then calculated for each subject by summing both components:

4 $$\:E{D}_{TOT}=E{D}_{CT}+E{D}_{PET-BMI}$$

Based on this calculation across all 47 subjects, the mean ± standard deviation of the total effective dose was (2.8 ± 0.2) mSv. A complete summary of subject-specific DLP values, injected activities, and resulting dose estimates is provided in Supplemental Table 1.

### Simulating lower PET activity levels

Dose reduction was simulated in two steps: (1) subsampling PET list-mode data to mimic lower activities, and (2) generating synthetic CTs for attenuation correction. First, the last 5 min (57–62 min p.i.) of list-mode PET data were extracted to create a “static” reference. The executable file “LMChopperStar_math.exe”, as part of the e7 tools and JSRecon12 (Siemens Healthineers), was used to reduce the number of events from the list-mode data to create four subsampled datasets simulating 50%, 25%, 10%, and 5% of the original [^18^F]FDG activity (~ 109 MBq, 100%). It employs the RAN3 algorithm, a subtractive random number generator with a long period and improved statistical properties, as described in Numerical Recipes [35]. Using the e7 tools (Siemens Healthineers), these datasets, along with the original list-mode with full 100% counts, were reconstructed to yield non-attenuation-corrected (NAC) PET images.

### CT-less PET image generation

**Fig. 2:**
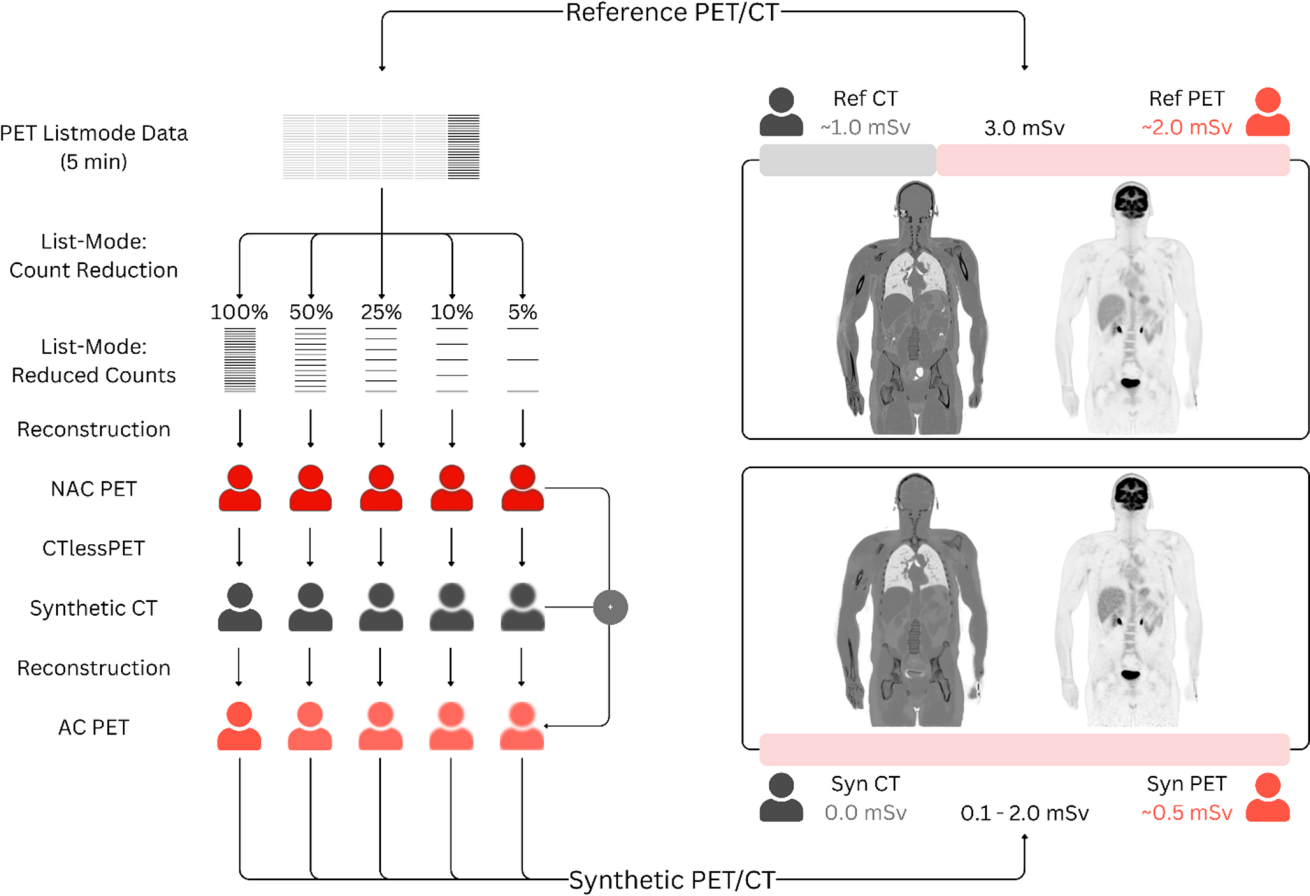
Concept of dose-dependent data generation. Counts were randomly removed from the static, 5-minute, list-mode data to simulate reduced-dose acquisitions. Non-attenuation-corrected (NAC) PET images were then reconstructed. Each NAC PET image was used as input for the software CTlessPET [24] to generate synthetic CT images for attenuation correction. The synthetic CTs, along with the reduced list-mode files, were used to reconstruct attenuation-corrected (AC) PET images at different dose levels

Synthetic CT images (synCTs) were generated using the software “CTlessPET”, which was used unmodified as presented by Montgomery et al. [24]. This software employs a conditional generative adversarial network to generate synthetic CT images at each dose level from the corresponding NAC PETs. These synCTs were then used in a second round of reconstructions with the subsampled list-mode data to produce attenuation-corrected PET images (synPETs). All reconstructions were performed with the e7 tools provided by Siemens Healthineers, using identical parameters to the reference PET: OP-OSEM with TOF/PSF, 4 iterations, 5 subsets, 440 × 440 × 531 matrix, voxel spacing 1.65 × 1.65 × 2 mm, 2 mm Gaussian post-filter, and standard corrections. The entire workflow is visualised in Fig. 2.

### Image analysis

To assess quantitative accuracy, volumes of interest (VOIs) were automatically derived from the refCTs using the segmentation software MOOSE [36]. Since the synthetic CT and PET images were generated based on the geometry of the original refPETs, which differs from the geometry of the refCTs, an initial alignment step of the generated VOI images was required. This alignment was performed using a global rigid (6-degree-of-freedom) centre transformation, followed by nearest-neighbour interpolation, to match the refCT geometry-based regions to the synthetic CT and PET image spaces for analysis. The analysis focused on bone marrow, liver, lungs, pancreas, spleen, thyroid glands, skeletal muscle, and subcutaneous fat at the L3 vertebral level [6].

For each VOI, Hounsfield Units (HU) were extracted from CT images, and mean body-weight-based Standardized Uptake Values (SUVbw) were calculated from PET images. This process allowed us to evaluate whether tissue-specific attenuation and metabolic characteristics were preserved in the synthetic datasets. The refCTs and the (57–62 min p.i.) refPETs served as references to assess the anatomical and quantitative accuracy of the synthetic datasets. Synthetic images were evaluated both through visual inspections for anatomical consistency and through voxel-wise percentage difference maps to identify regions with high discrepancies (>10% threshold). Visual inspection extended to peripheral regions (brain and lower extremities), known to be underrepresented in the CTlessPET training dataset [24]. Percentage differences between synthetic and reference images were calculated on an organ basis as:

5 $$\:{I}_{{\Delta\:}\mathrm{\%}}=100\times\:\frac{{I}_{syn}-{I}_{ref}}{({I}_{syn}+{I}_{ref})/2}$$

where $\:{I}_{syn}$ and $\:{I}_{ref}$ are the mean voxel intensity values of the synthetic and reference images, respectively, per organ. The same formula was applied to mean values within each VOI (with both SUVbw and HU). Absolute percentage differences were reported unless stated otherwise.

Deviations from the reference quantities were assessed at both group and subject levels. For the group analysis, the mean HU and SUVbw were calculated across the entire cohort for each VOI and simulated dose level. These group averages were then systematically compared to the corresponding reference values obtained from the original standard images. At the individual subject level, the absolute differences in extracted quantitative parameters between the reference images and each synthetic image at progressively reduced dose counts were computed for each participant. These subject-specific deviations were then averaged across the entire cohort.

Prior to statistical comparisons, we evaluated data distribution characteristics using the Shapiro–Wilk normality test. Since not all variables met the assumption of normal distribution (*p* < 0.05), we implemented non-parametric equivalence testing. For the group-level comparisons between synthetic and reference data, we employed the Mann-Whitney Two One-Sided Test (TOST) procedure with a conservative equivalence margin set at 5% of reference values. Subject-level comparisons utilised the Wilcoxon signed-rank test for paired analyses. In all statistical tests, we maintained a significance threshold of *p* < 0.05. All statistical comparisons were performed pairwise between reference and synthetic datasets at each dose level. Since each analysis was restricted to two datasets at a time, no global set of simultaneous hypotheses was tested. Accordingly, we did not apply any statistical correction for multiple comparisons, and unadjusted p-values for each organ- and subject-level comparison are reported.

For image noise evaluation, we extracted the coefficient of variation (CV) from a standardised 3 cm diameter spherical VOI placed at the liver centroid, as recommended by the EANM guidelines for assessing PET image quality [37]. The liver is typically used for this purpose because it represents a large, homogeneous organ with relatively stable FDG uptake and central positioning in the field of view. Using a small, centrally placed VOI instead of the entire liver volume also minimises potential quantification errors arising from respiratory motion and misalignment at organ boundaries. CV values below 15% were considered clinically acceptable [38]. The total effective dose for each simulated protocol was computed by summing the null dose contribution from synthetic CT (0 mSv) with the progressively lowered PET component doses derived from our subsampling approach.

## Results

A total of 47 healthy volunteers (25 females, 22 males) were included in the analysis (Fig. 1A). Based on BMI classification, most participants were of normal weight (61.7%), with smaller proportions in the underweight (2.1%), overweight (31.9%), and obese categories (4.3%) (Fig. 1B). The mean age of the cohort was 38 ± 14 years, with a comparable distribution between males (38.5 ± 12.1 years) and females (37.5 ± 15.2 years) (Fig. 1C). The injected [^18^F]FDG activity averaged 109 ± 7 MBq across all subjects, with no significant differences between sexes.

**Fig. 3:**
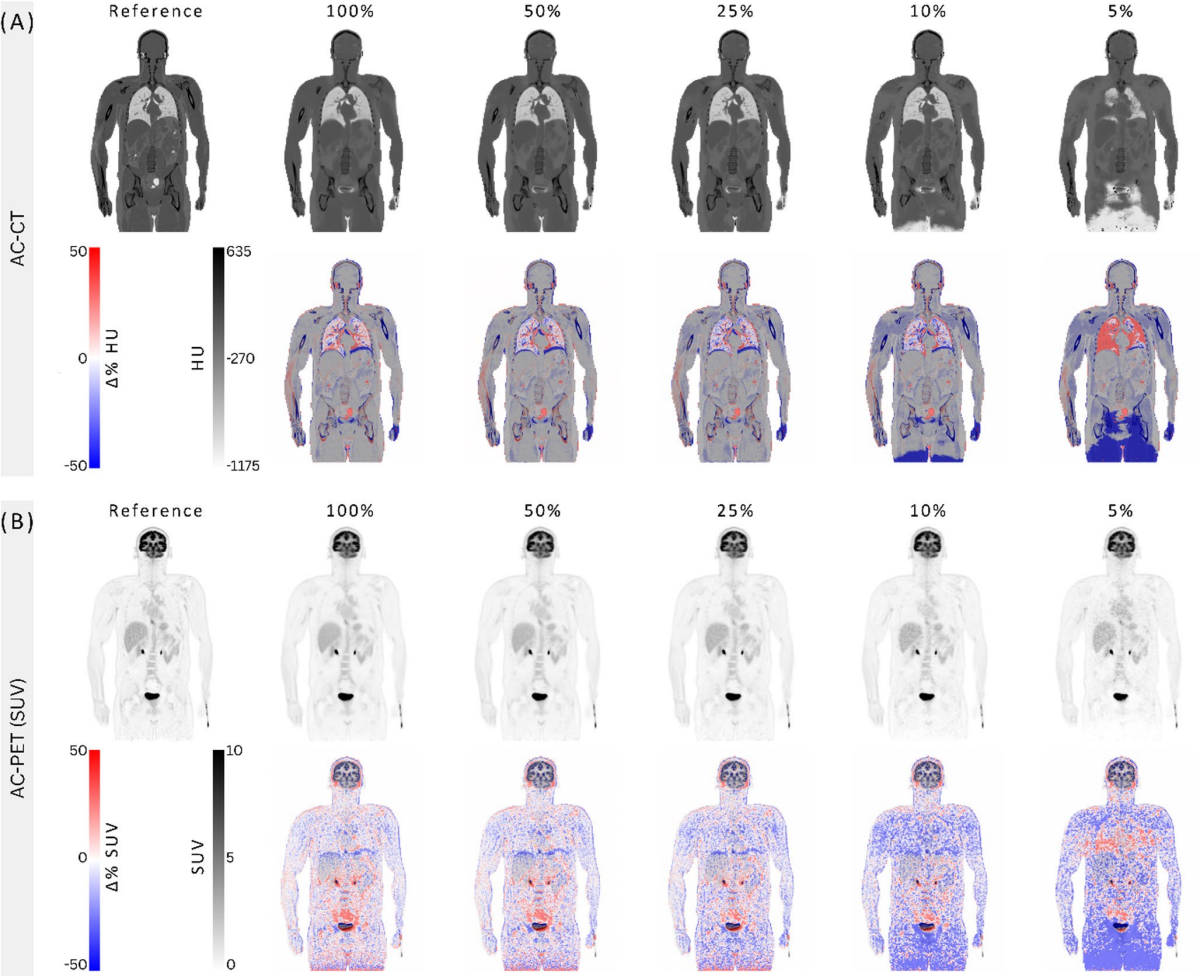
AC-CTs and AC-PET (SUV) images at different dose levels for a healthy subject (ID #002, male, 40 years, BMI = 20.6 kg/m²). (**A**) Reference CT and synthetically generated CTs from CTlessPET [24] at five dose levels next to the original (reference) CT (first row), with %-difference overlays relative to the reference CT (second row). (**B**) The first row shows the reference and synthetic PETs (reconstructed using CTs from (**A**)). The second row shows %-difference overlays relative to the reference PET

**Fig. 4:**
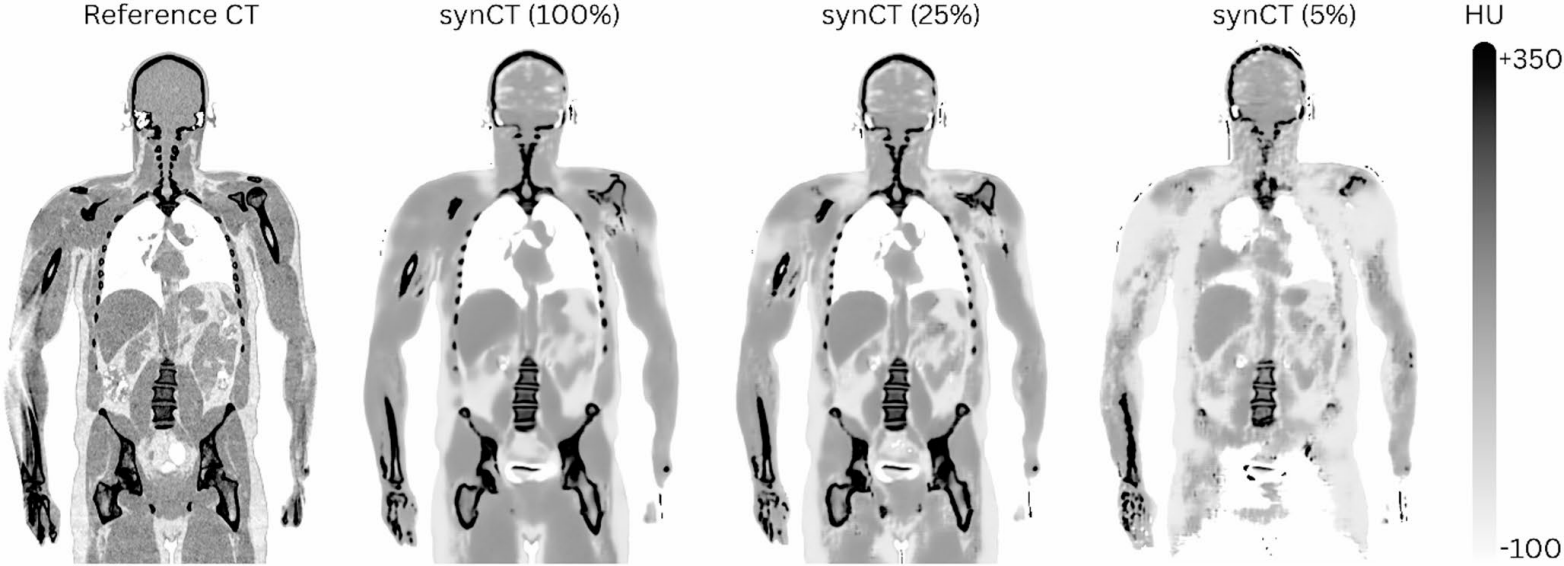
Subject #002 (Male, 40-years, BMI = 20.6 kg/m²). Comparison between the reference CT and the corresponding synthetic CTs (synCT) reconstructed at 100%, 25%, and 5% of the original counts. As the count level decreases, the synCTs show progressive degradation in contrast and anatomical detail, with substantial blurring and loss of fine structures evident across the entire image at 5%. Tissue boundaries, especially between skeletal muscle and adipose tissue, become less distinct, and overall image quality is markedly compromised at the lowest dose level

Visual inspection revealed close general anatomical correspondence between synCTs and refCTs across all activities (Fig. 3A). At 100% and 50% counts, only minimal deviations (< 10%) were observed in regions not affected by motion-induced PET-CT misalignment, while higher deviations were seen in the lower lung-upper abdomen transition and in the extremities. Progressive blurring of the synCTs became apparent at 25% counts, particularly at soft tissue interfaces (such as in the abdominal regions), with more pronounced loss of fine structural details and poor delineation of the abdominal structures at 10% and 5% counts (Fig. 4). The %-difference maps confirm this trend, showing maximal deviations (up to 50%) at lower doses and tissue boundaries, especially the lung edges. Significant differences also occurred at the imaging field edges (Fig. 3A), including the head and legs (Fig. 4).

SynPET images were similar to the refPET images at all dose levels, though differences increased slightly as the simulated counts, or activity levels, were reduced. The liver dome consistently showed SUVbw deviations up to −25% in synPET versus refPET (Fig. 3B).

**Fig. 5:**
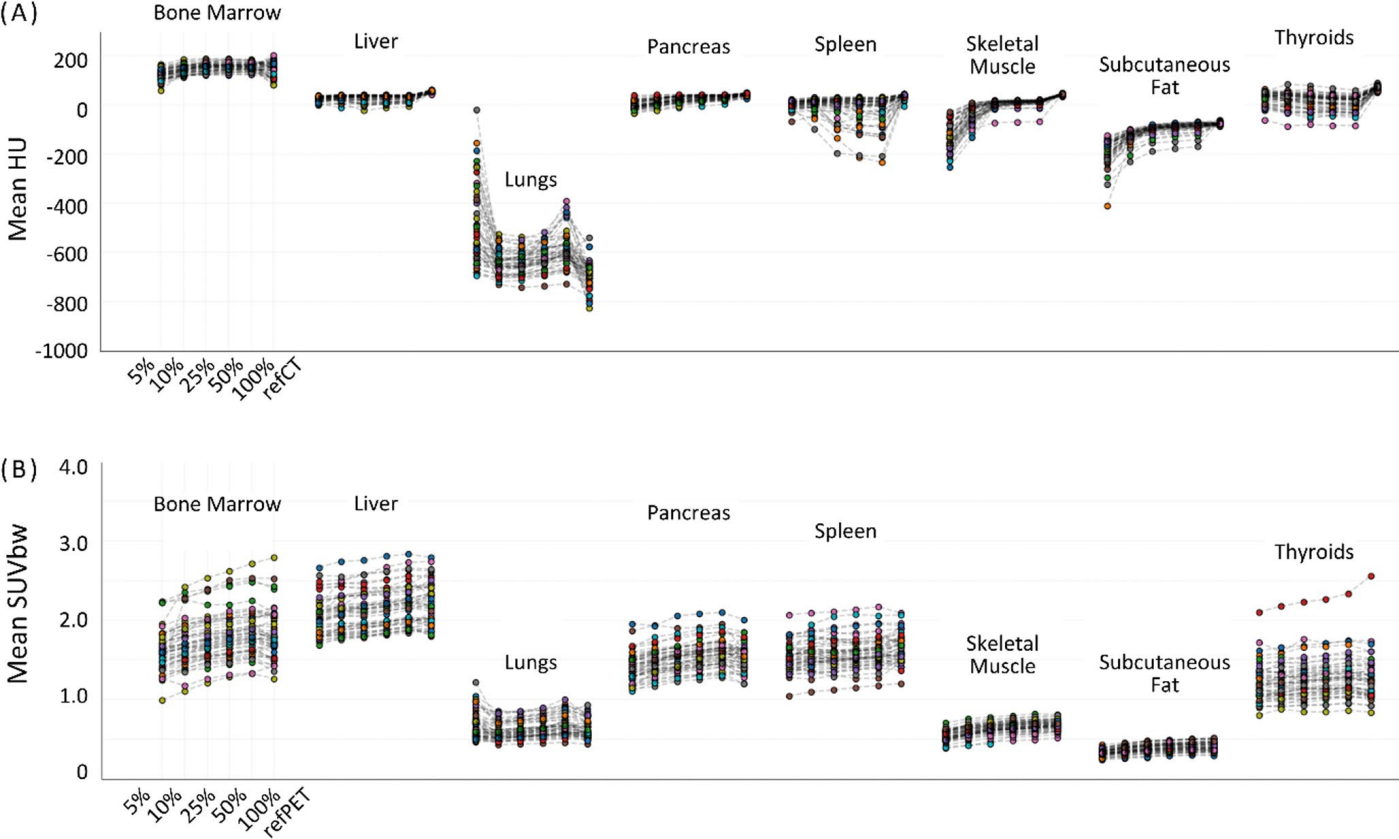
(**A**) Mean HU per region across all subjects for the synthetic CT images at 5, 10, 25, 50, and 100% dose with the reference CT (refCT) as the reference point. (**B**) Mean SUVbw per region across all subjects for the dose-reduced PET images at 5, 10, 25, 50, and 100% dose with the reference PET (refPET) as the reference point

When comparing synCT and synPET metrics against their respective reference images, cohort-level analysis demonstrated substantial variability in mean Hounsfield Units across all selected tissues in the synCT images (Fig. 5A), indicating inconsistent tissue density preservation during the generation of the synthetic CT images. However, synPET and refPET exhibited strong mean SUVbw agreement across all organs and dose levels (Fig. 5B).

Group level Mann–Whitney TOST analysis showed no significant differences between the synPET and the refPET at 100% dose level for any VOI (*p* > 0.05, Table 1). As dose decreased, mean SUVbw deviations increased slightly, but remained within < 10% down to 10% dose, reaching significance only in lungs at 5% counts (*p* = 0.03).

**Table 1:** Group-level mean SUVbw variations per target volume computed from the synthetic PET images at each reduced count level and compared to the reference PET. Differences are reported as group averages, with statistical significance assessed using a Mann-Whitney-based TOST (with 5% difference threshold) between the reference PET and the 100% synthetic PETs. Significant differences are highlighted in bold. STD = standard deviation

	Reference PET	%Differences: synthetic PET (5 count levels) vs. reference PET				
Target Volume	mean SUV ± STD	5%	10%	25%	50%	100%
Bone Marrow	1.8 ± 0.3	12% *p* = 0.29	6% *p* = 0.71	4% *p* = 0.73	< 1% *p* = 0.78	2% *p* = 0.73
Liver	2.2 ± 0.2	7% *p* = 0.45	5% *p* = 0.75	5% *p* = 0.80	2% *p* = 0.78	< 1% *p* = 0.80
Lungs	0.6 ± 0.1	10% ***p*** **= 0.03**	4% *p* = 0.64	2% *p* = 0.11	3% *p* = 0.40	9% *p* = 0.11
Pancreas	1.5 ± 0.2	9% *p* = 0.52	4% *p* = 0.86	2% *p* = 0.57	1% *p* = 0.61	4% *p* = 0.57
Skeletal Muscle	0.6 ± 0.1	15% *p* = 0.24	8% *p* = 0.62	6% *p* = 0.83	2% *p* = 0.80	< 1% *p* = 0.83
Spleen	1.6 ± 0.2	8% *p* = 0.46	5% *p* = 0.76	6% *p* = 0.69	4% *p* = 0.68	2% *p* = 0.69
Subcutaneous Fat	0.4 ± 0.1	14% *p* = 0.45	8% *p* = 0.72	6% *p* = 0.85	2% *p* = 0.83	< 1% *p* = 0.86
Thyroids	1.3 ± 0.3	5% *p* = 0.40	3% *p* = 0.70	2% *p* = 0.84	< 1% *p* = 0.79	2% *p* = 0.76

Subject-level Wilcoxon test showed greater variability, with significant differences between the synPET and the refPET even at 100% counts in bone marrow, lungs, pancreas, and thyroid (Table 2). Despite this, absolute deviations generally stayed below 10%, except in skeletal muscle at 5% (= 15%).

**Table 2:** Subject-specific variability of mean SUVbw was assessed for each target volume by comparing synthetic PET images at reduced count levels to the reference PET. Differences were calculated as the average of mean absolute percentage differences per subject. Statistical significance was evaluated using a Wilcoxon signed-rank test between the reference PET and the synthetic PETs. Significant differences are indicated in bold. STD = standard deviation

	Reference PET	%Differences: Synthetic PET (5 count levels) vs. reference PET				
Target Volume	mean SUV ± STD	5%	10%	25%	50%	100%
Bone Marrow	1.8 ± 0.3	12% ***p*** **< 0.01**	7% ***p*** **= 0.02**	6% *p* = 0.90	4% *p* = 0.11	5% ***p*** **= 0.01**
Liver	2.2 ± 0.2	7% ***p*** **< 0.01**	5% ***p*** **= 0.02**	5% ***p*** **= 0.01**	3% ***p*** **= 0.03**	3% *p* = 0.81
Lungs	0.6 ± 0.1	13% ***p*** **< 0.01**	8% *p* = 0.43	8% *p* = 0.57	7% ***p*** **< 0.01**	11% ***p*** **< 0.01**
Pancreas	1.5 ± 0.2	10% ***p*** **< 0.01**	6% *p* = 0.75	6% ***p*** **= 0.03**	5% ***p*** **< 0.01**	6% ***p*** **< 0.01**
Skeletal Muscle	0.6 ± 0.1	15% ***p*** **< 0.01**	9% ***p*** **< 0.01**	7% ***p*** **= 0.01**	4% *p* = 0.64	4% *p* = 0.59
Spleen	1.6 ± 0.2	8% ***p*** **< 0.01**	5% *p* = 0.05	6% *p* = 0.07	5% ***p*** **= 0.02**	5% *p* = 0.05
Subcutaneous Fat	0.4 ± 0.1	14% ***p*** **< 0.01**	9% ***p*** **< 0.01**	7% ***p*** **= 0.03**	5% *p* = 0.98	5% *p* = 0.38
Thyroids	1.3 ± 0.3	5% ***p*** **< 0.01**	4% ***p*** **< 0.01**	4% *p* = 0.05	3% ***p*** **< 0.01**	4% ***p*** **< 0.01**

**Fig. 6:**
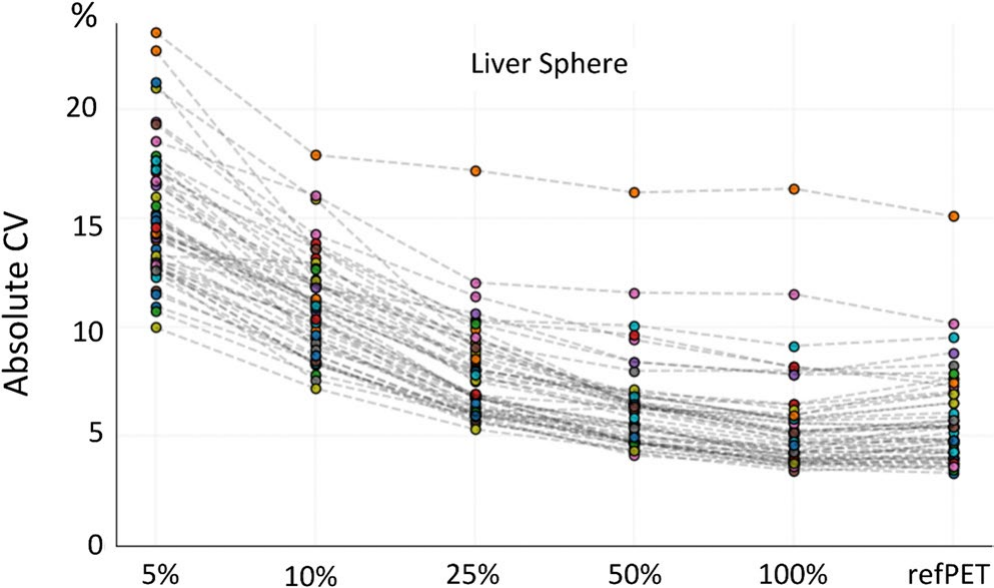
Coefficient of Variations (CV) across all subjects for the dose-reduced PET images at 5%, 10%, 25%, 50%, and 100% dose, and the reference PET (refPET) as the reference point. CV was extracted from a spherical VOI placed in the liver. The outlier showing CV > 15% at all dose levels presented high motion artefacts (see Fig. 7)

The quality of the synthetic PET images declined with reduced activity levels, yet the CV remained within the commonly accepted threshold of 15% [38] in 46 out of 47 subjects, down to 25% of the original counts (Fig. 6). This corresponded to an average effective dose per subject of (0.45 ± 0.04) mSv, which represents approximately 16% of the reference total effective dose per subject (2.8 ± 0.2 mSv) (Table S1).

## Discussion

Using a cohort of 47 healthy Caucasian controls [25], we demonstrate the feasibility of combining total-body [^18^F]FDG-PET/CT imaging with synthetic CT-based attenuation correction for quantitative organ-level analysis at minimal dose regimens. By reducing the injected [^18^F]FDG activity to ~ 25 MBq and employing a previously published approach to generate synthetic CT images, we achieved a total effective dose of approximately 0.45 mSv (versus 2.8 mSv reference) while maintaining organ-based PET metrics compared to the original PET/CT protocol.

Although our baseline protocol of 2.8 mSv was already below published DRLs (typically 10–20 mSv for whole-body [^18^F]FDG-PET/CT in adults [13, 14]), further reduction to 0.45 mSv may provide added value in research and screening contexts: it minimises cumulative exposure in repeated studies, improves acceptability for healthy volunteer cohorts, and strengthens compliance with optimisation principles, especially given that, unlike workers and the general public, patients in the EU are not subject to fixed annual dose caps. Instead, medical exposures are governed by justification and optimisation, with DRLs serving as non-binding reference benchmarks. In this light, further lowering already low doses can be important for future preventive imaging or normative atlas creation.

Several CT-free attenuation strategies have been reported, including direct mappings from NAC-PET to AC-PET [39, 40], estimation of synthetic AC-PET and CT/µ-maps from NAC-PET [41], and sinogram- as well as image-domain approaches, often TOF-based, that estimate attenuation from emission data in brain imaging [42, 43]. We selected the CTlessPET method of Montgomery et al. [24] because it generates an explicit synCT that can be visually inspected and used within a standard reconstruction workflow, operates on routinely available NAC images without requiring vendor-specific raw-data access, and has been validated on Siemens Biograph Vision–family TB datasets aligned with our acquisition protocol.

The synthetic CTs provided sufficient structural information for basic anatomical localisation and attenuation correction, thereby eliminating the need for a separate, conventional CT scan. However, they could not match the diagnostic resolution of standard CT scans, particularly in low-dose conditions (Fig. 4), thus limiting a wider clinical adoption in cases where accurate anatomical interpretation is needed. Nonetheless, in scenarios where such high-quality diagnostic CT is not required, such as in screening studies, selected follow-up examinations, or opportunistic metabolic imaging of healthy controls, such synthetic CT is of use. Naturally, in clinical settings that require high-resolution tissue characterisation, omitting a diagnostic CT would be inappropriate. A hybrid approach, using synthetic CT initially with selective diagnostic CT for suspected findings, could optimise radiation exposure without compromising diagnostic accuracy.

The visual quality of the synthetic CT showed direct dependence on PET dose levels (Fig. 4). At the lowest simulated doses (5%–10%), the synCTs appeared significantly blurred, particularly at soft-tissue interfaces. This degradation likely results from inherent smoothing effects in the image synthesis process, and physiological motion, especially respiration, captured during PET acquisition and embedded in the NAC PET input. Most likely, both factors contribute to the poor structural delineation observed in low-dose reconstructions (Fig. 3A). Inaccuracies at the edges of the synCTs field-of-view were also noted, particularly in the lower extremities and head regions. These peripheral discrepancies may reflect the anatomical under-representation in the training dataset of the CTlessPET model, which was not specifically optimised for legs or brain anatomy, as stated by the authors [24]. This effect is very noticeable in the head region (Fig. 4), where even at 100% counts, the synthetic CT failed to reproduce fine anatomical details. Despite these limitations, PET reconstructions using synthetic CTs maintained both visual and quantitative robustness down to 25% of the original injected activity, with clinically acceptable image noise levels (CV < 15%) [38] across 46 out of 47 subjects (Fig. 6).

The synthetic CTs showed a markedly high variability in Hounsfield Units across different dose levels, particularly in the lung tissues (Fig. 5A**)**. These absolute differences were diminished in parts by the transformation of Hounsfield units into linear attenuation coefficients at 511 keV [44], and, therefore, subsequent PET-derived SUVbw – post synCT-based attenuation correction - remained stable across dose levels (Fig. 5B). This can also be attributed to the fact that CT-based attenuation correction factors (ACF) are calculated along a given line-of-response across multiple voxels; here, an integration over many noisy voxel values results in a less noisy value of the total ACF, and, thus, attenuation-corrected PET value.

At the group level, we found no statistically significant differences between synthetic PET and reference PET in most VOIs (Table 1), with average differences remaining below 10% across all organs. However, intra-subject variability analysis revealed more pronounced variability when comparing synthetic PETs at different dose levels to the reference PET. This suggests that while population averages remain stable, individual-level deviations may occur with dose reduction and synthetic CT usage. This effect was especially evident in tissues with inherently variable metabolism, such as bone marrow, pancreas, and thyroid (Table 2).

**Fig. 7:**
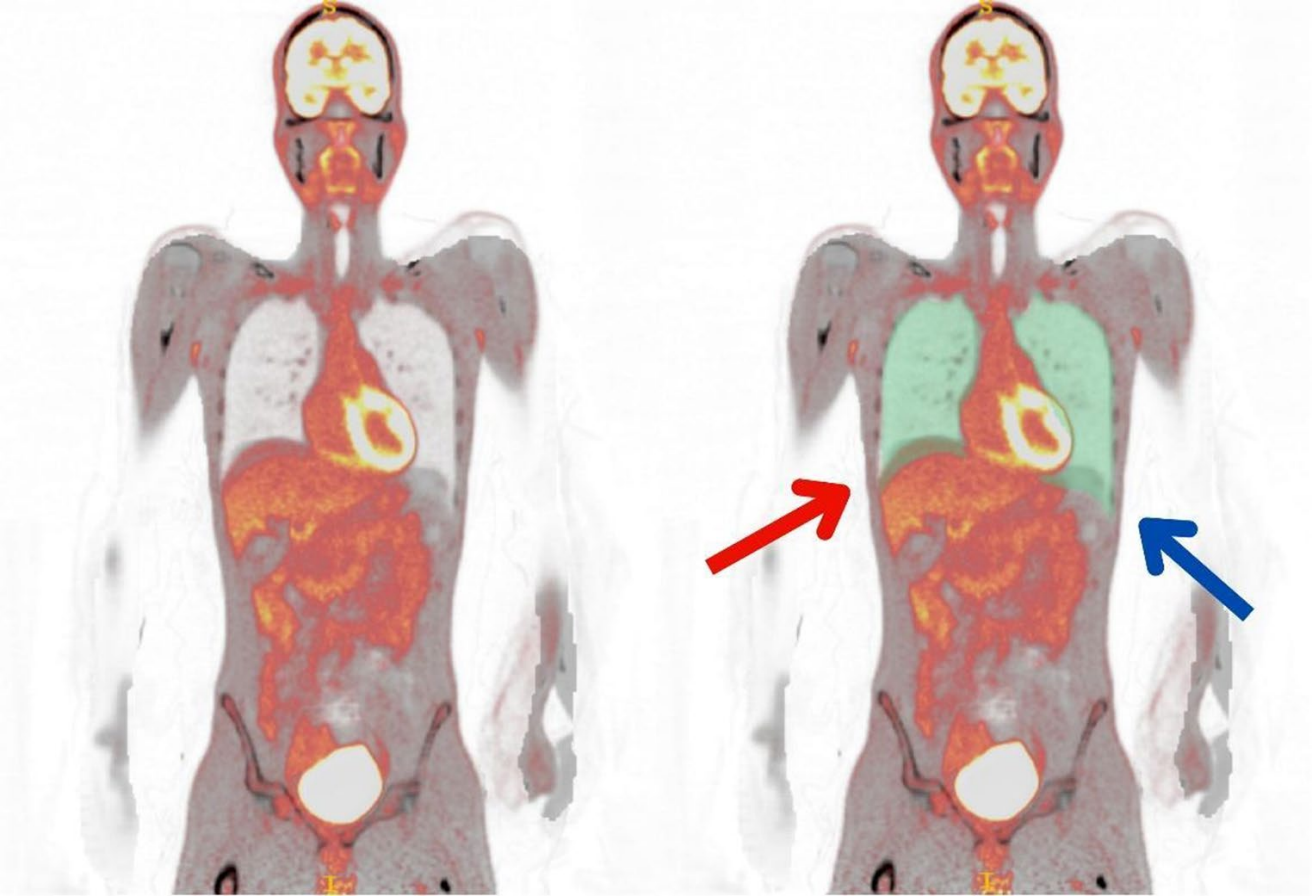
Subject #031 (Male, 32-years, BMI = 19.0 kg/m²). Example of overlapping reference PET and CT images showing significant motion-induced misalignment. Breathing motion artefacts are visible near the dome of the liver (red arrow) and the spleen (blue arrow). As a result, the lung segmentation, automatically derived from the reference CT, does not align perfectly with the PET image. Motion-induced misalignment is also evident in the shoulder and arm regions

Some of the observed intra-subject variabilities may relate to the segmentation quality. Our workflow used automatic segmentations generated from the reference CTs, which were then overlapped with the synthetic reconstructions. This approach depends critically on precise PET/CT alignment. Any intra-scan subject motion, such as breathing, could lead to inaccurate VOI placement and biased quantification (Fig. 7). To improve intra-subject consistency, one solution would be to employ data-driven gating (DDG) [45] that was not available on our system at the time of this study. Another post-acquisition solution is to shrink the segmented VOIs and reduce their volume to minimise edge misclassification.

This study had several limitations. The segmentation software MOOSE [36] used in our pipeline requires a structural image (i.e., low-dose CT) as input. It performed suboptimally when applied directly to synthetic CTs, given their low image contrast and lack of quantitative image information. Of note, MOOSE itself was trained on CT data that were acquired with an effective dose of 0.5–1.5 mSv. If the ultimate goal is the complete CT elimination, either PET-based segmentation tools must be developed, or segmentation tools must be trained explicitly on synthetic CT for this purpose. Further technical limitations include the fact that CTlessPET [24] was trained using data from patients scanned with arms-up positioning, whereas our study used arms-down acquisition. This discrepancy likely introduced artefacts in the upper body region. Moreover, the model was not optimised for head coverage, which may explain the visual inaccuracies observed at the upper edge of the field of view. These observed artefacts and inaccuracies might be further amplified by the fact that the original training data for CTlessPET were acquired on a different scanner within the same Biograph Vision family as ours, despite the close hardware lineage. Our evaluation is also confined to [^18^F]FDG; other tracer-specific uptake patterns could affect the generalizability of our results. Future studies should aim to extend and assess the approach for additional radiotracers by potentially applying the transfer-learning strategy described by Montgomery et al. [24]. Another limitation concerns the simulation of reduced [^18^F]FDG activities by random list-mode subsampling. While this approach is commonly used to approximate lower-count conditions, it does not fully reproduce the different scaling behaviours of true and random coincidences, since the true count rate increases approximately linearly with activity, whereas the randoms scale quadratically [46]. Additional nonlinearities related to dead time and pile-up losses further complicate a simple proportional down-scaling of the acquired prompts [46]. In our study, the randoms fraction was comparatively small at the administered activity levels (~ 100 MBq) and under ultra-high sensitivity acquisition conditions, such that the bias introduced by ignoring these effects is expected to be minor. Nonetheless, this approximation should be acknowledged as a potential source of error, especially if the method were to be extended to higher activity levels or to scanners with different randoms characteristics.

Further, we did not adjust the PET image reconstruction (e.g., fewer iterations, larger voxel sizes and stronger filtering) to account for lower activity levels [47]. In addition, it can be assumed that increasing the emission time window from 5 min to 10 min (at 60 min p.i.) helps improve noise characteristics of the resulting emission images, and, thus, may help to further lower the injected tracer activities. Finally, our study focused specifically on organ-based analysis; more extensive data collection and evaluation would be needed to assess performance for lesion quantification or voxel-based analyses.

## Conclusions

Our proposed low-dose imaging protocol combines TB-[^18^F]FDG-PET with synthetic CT-attenuation maps (synCT) to lower effective subject radiation exposure to 0.45 mSv. The primary application of this protocol is in research settings for establishing normative metabolic activity in healthy control populations. Furthermore, it is well-adapted for low-dose screening and surveillance programs where positive findings could be referred for confirmatory, high-quality PET/CT. This protocol is not recommended for clinical scenarios demanding high spatial resolution, such as the detection and characterisation of small lesions, given the inherent limitations in spatial resolution of synCT.

## Supplementary Information

Below is the link to the electronic supplementary material.

Supplementary Material 1

## Data Availability

The dataset used as reference images for analysis and comparison with the synthetic images at reduced dose levels can be accessed on Zenodo via this link: https://zenodo.org/records/16364694 [25].

## Code availability

CTlessPET was used as published in [24], MOOSE as in [36] with both being available via https://github.com/DEPICT-RH/CTlessPET and https://github.com/ENHANCE-PET/MOOSE, respectively. Analysis scripts are available on reasonable request.
