# Supplementary material for: Total-body [18F]FDG-PET/CT imaging of healthy volunteers with minimal effective dose
Source: Eur J Nucl Med Mol Imaging. 2025 Nov 18;53(4):2622–35. doi: 10.1007/s00259-025-07644-x (PMC12920338; doi:10.1007/s00259-025-07644-x)
Supplement: Supplementary file 1 — Supplementary Material 1 [file 259_2025_7644_MOESM1_ESM.docx]

**Sebastian Gutschmayer, MSc**
QIMP Team, AKH Wien, Leitstelle 4L
Waehringer Guertel 18-20
1090 Vienna, Austria
Phone: +43-1-40400-39790
Email: sebastian.gutschmayer@meduniwien.ac.at

**September 12, 2025**

**Subject: Manuscript submission of
*“Total-body [18F]FDG-PET/CT imaging of healthy volunteers with minimal effective dose”***

Dear Professor Carrio, MD,

On behalf of my co-authors, I am pleased to submit our manuscript entitled “**Total-body [18F]FDG-PET/CT imaging of healthy volunteers with minimal effective dose**” for consideration for publication in the *European Journal of Nuclear Medicine and Molecular Imaging*.

Our study explores the feasibility of combining total-body [18F]FDG-PET with reduced [18F]FDG activity and deep learning–based synthetic CT attenuation correction to significantly reduce effective radiation dose in healthy subjects. We demonstrate that PET imaging can maintain quantitative integrity at effective doses as low as ~0.45 mSv, thereby supporting the creation of normative datasets for systemic metabolic imaging while adhering to the ALARA principle. We believe these findings will be of interest to the readers of *EJNMMI*, as they address a growing interest in minimising effective radiation exposure for PET/CT imaging study participants, especially in view of the emergence of AI composite tools and long axial field-of-view PET systems.

**Authorship note**: We would also like to draw your attention to the first authorship of this manuscript. Daria Ferrara and Sebastian Gutschmayer, as early-stage researchers in this group of authors, are listed as equally contributing first authors for their central roles in the conceptualisation of this study, data collection, and jointly performing the quantitative analyses.

All authors have approved the final version of the manuscript, and the work has not been published or is under consideration elsewhere. We have disclosed all relevant funding and potential competing interests within the manuscript.

We thank you for considering our work for publication in *EJNMMI*. We look forward to your feedback.

Sincerely,

**Sebastian Gutschmayer, MSc**
(on behalf of all authors)
